# Supplementary material for: BMP9 stimulates joint regeneration at digit amputation wounds in mice
Source: Nat Commun. 2019 Feb 5;10:424. doi: 10.1038/s41467-018-08278-4 (PMC6363752; doi:10.1038/s41467-018-08278-4)
Supplement: Supplementary file 1 — Supplementary Information [file 41467_2018_8278_MOESM1_ESM.pdf]

BMP9 stimulates joint regeneration at digit amputation wounds in mice

Yu, Dawson et al.

Supplementary Materials:

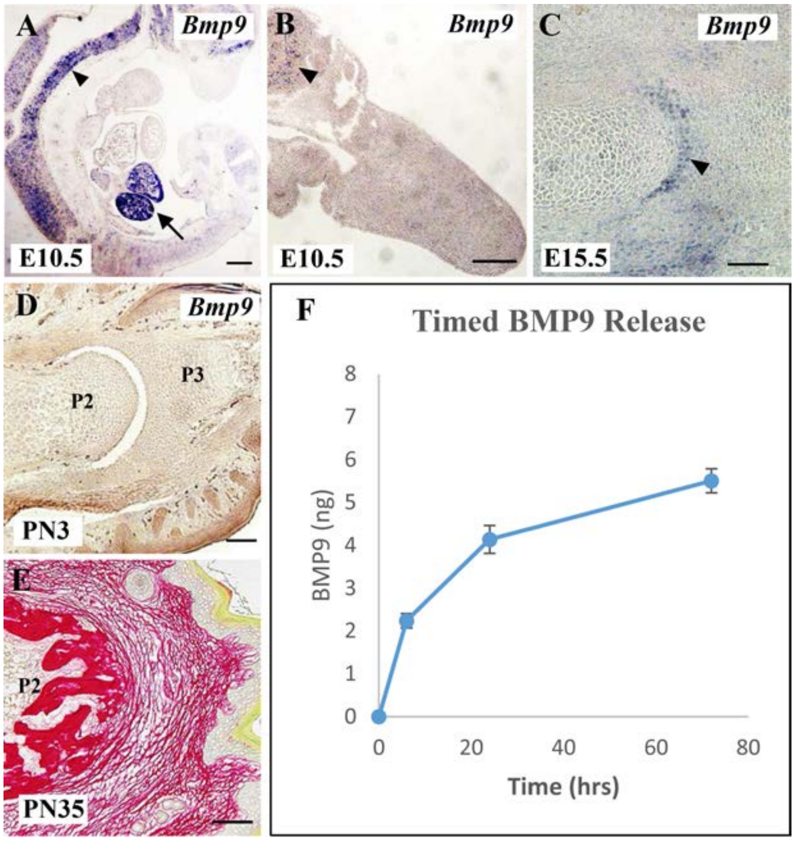

Supplementary Fig. 1 a) In situ hybridization of a mid-sagittal section of an E10.5 embryo showing prominent expression of *Bmp9* in the liver (arrow) and central nervous system (arrowhead). **b**) In situ hybridization of a cross section of an E10.5 embryo at the level of the limb bud showing *Bmp9* expression in the spinal cord (arrowhead) but no expression in the limb bud. **c**) In situ hybridization identifies *Bmp9* transcripts expressed in the joint forming regions of the E15.5 digit. **d**) In situ hybridization of the PN3 digit showing the absence of *Bmp9* transcripts of the P2/P3 joint. **e**) Picrosirus red staining of a non-regenerative neonatal P2 amputation showing a bone stump capped by fibrous connective tissues. **f**) Timed release of BMP9 from a single agarose bead over 72 hours in vitro. A total of 5.5ng of BMP9 is released with the majority released during the first 24 hours. Data represent mean ± s.d. Scale bars: **a**=200µm; **b-e**=100µm

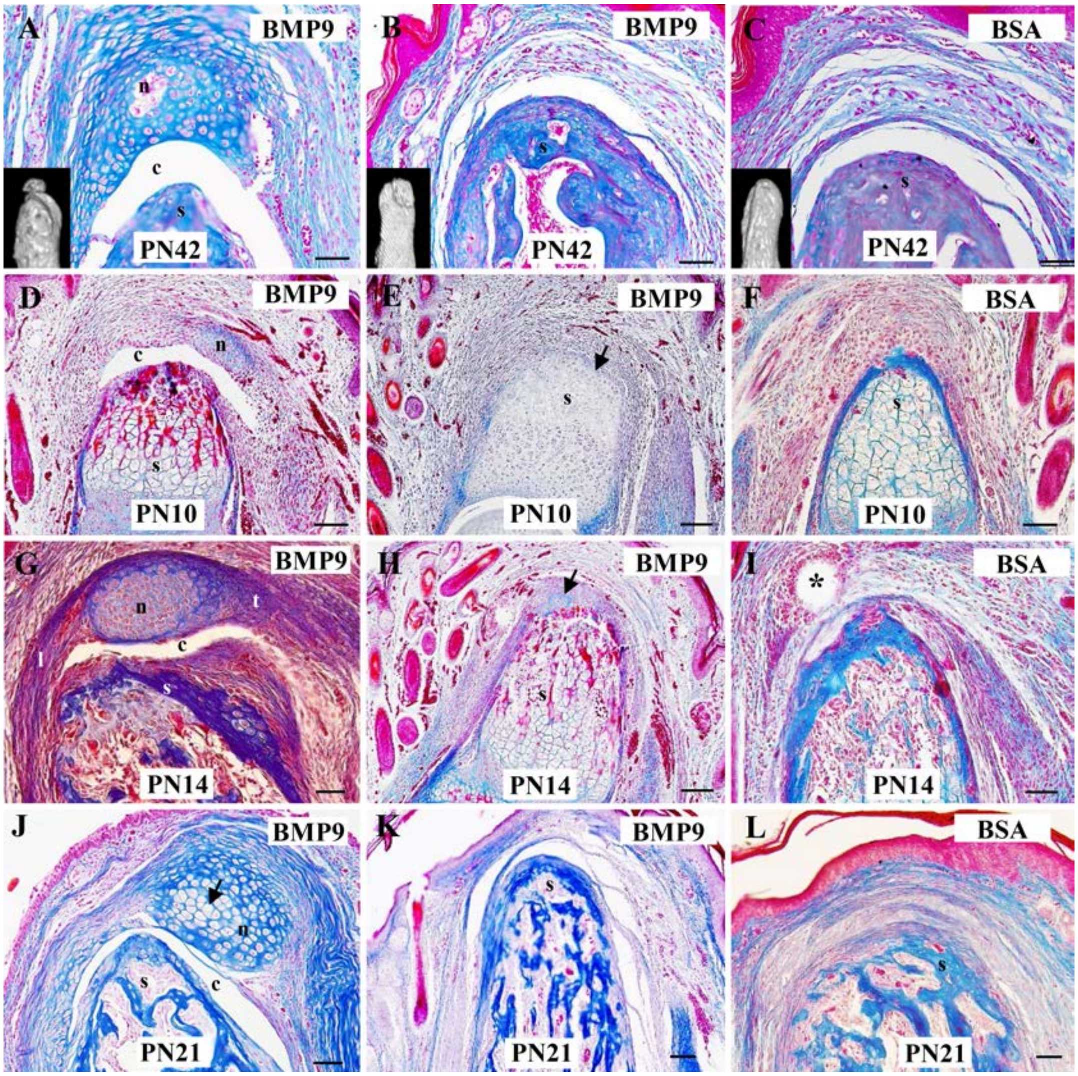

Supplementary Fig. 2 Mallory trichrome staining of BMP9- and BSA-treated digits at varying times after bead (*) implantation (s, stump; n, nodule; c, cavity). **a**) 5 weeks after BMP9 treatment (PN42) µCT rendering (inset) identifies a cavity forming regenerate that consists of a small distal skeletal nodule surrounded by chondrogenic tissue and separated from the stump by a well-defined cavity. **b**) 5 weeks after BMP9 treatment (PN42) µCT rendering (inset) identifies a non-cavity forming digit that consists of a healed stump covered by fibrous connective tissue. **c**) 5 weeks after BSA treatment (PN42) µCT rendering (inset) identifies a healed bony stump covered by fibrous connective tissue. **d**) 3 days after BMP9 treatment some digits form a well-defined cavity separating the stump from a regenerated distal chondrogenic nodule. **e**) 3 days after BMP9 treatment, non-cavity forming digits have chondrogenic cells (arrow) associated with the distal end of the stump. **f**) 3 days after BSA treatment, mesenchymal cells fill the amputation wound. **g**) Cavity forming digits 7 days after BMP9 treatment maintain a well-defined cavity separating the stump from a regenerated distal chondrogenic nodule. At this stage the regenerated nodule has well-defined connections with fibrous tissues that tract to the ventral tendon (t) and dorsal ligament (l) of the stump. **h**) Non-cavity forming digits 7 days after BMP9 treatment heal with an aggregation of cells (arrow) on the distal stump. **i**) 7 days after BSA

treatment fibrous tissue is associated with the distal stump. **j**) Cavity forming digits 14 days after BMP9 treatment maintain a well-defined cavity separating the stump from a regenerated distal chondrogenic nodule. At this stage hypertrophic chondrocytes are present in the center of the regenerated nodule (arrow). **k**) The digit stump of non-cavity forming digits 14 days after BMP9 treatment is differentiated with no sign of chondrogenic cells. **l**) The digit stump 14 days after BSA treatment forms a covering of fibrous tissue associated with the healed bone. Distal is toward the top and dorsal is to the left. Scale bars: **a**-**c**, **l**=50µm; **d**-**k**=100µm

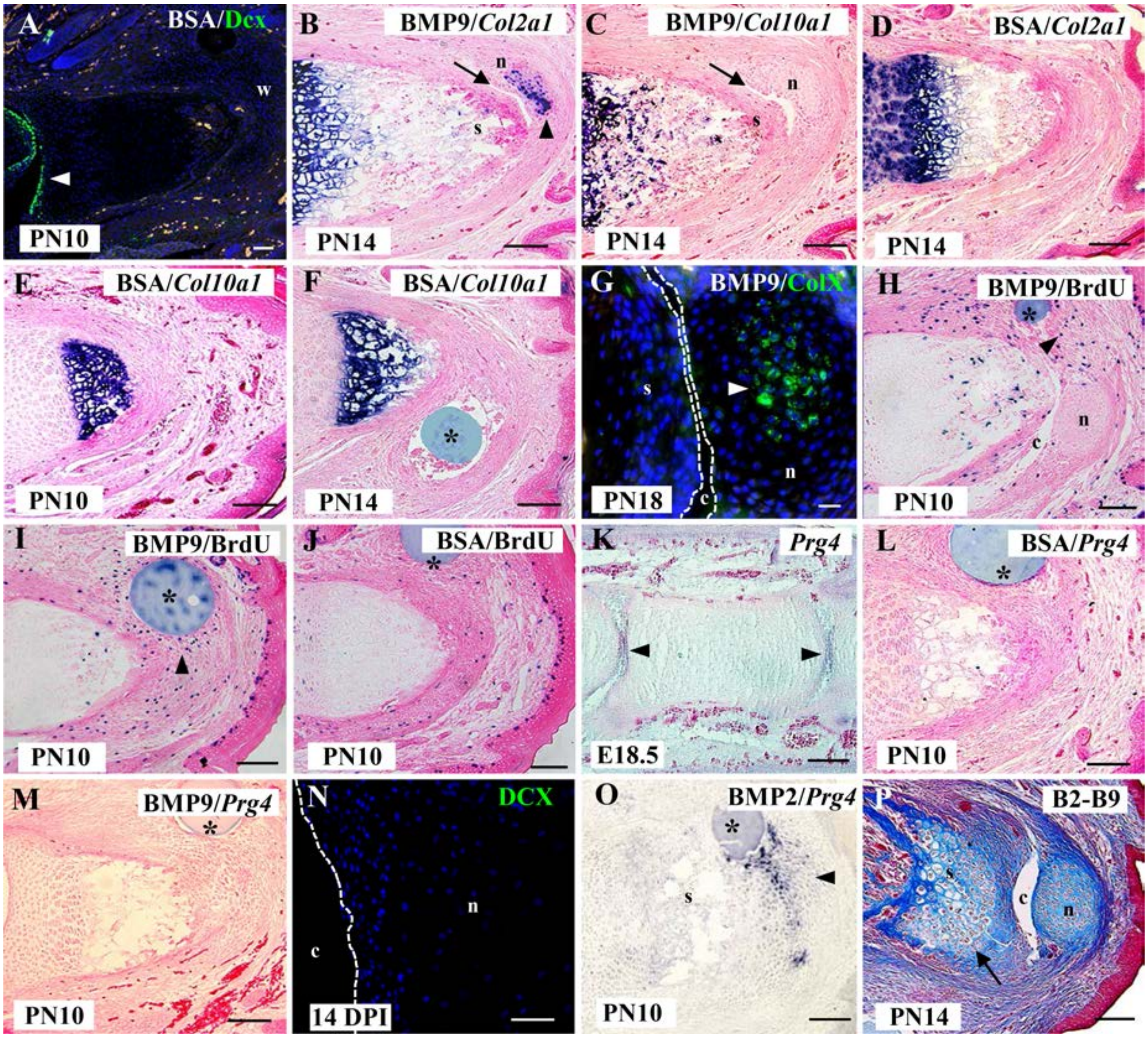

Supplementary Fig. 3 a) Immunostaining of BSA-treated control digits at 72 hours shows Dcx+ cells associated with the proximal P2 growth plate (arrowhead) and no staining of cells in the amputation wound (w). **b**-**f**) In situ hybridization studies of *Col2a1* and *Col10a1* expression following BMP9 or BSA bead (*) treatment (cavity, arrow; n, nodule; s, stump). **b**) In cavity forming digits 7 days after BMP9 treatment, the induced nodule is positive for *Col2a1* (arrowhead) but the stump is negative. **c**) In cavity forming digits 7 days after BMP9 treatment, the induced nodule and the stump are negative for *Col10a1*. **d**) Amputation wound cells do not up-regulate *Col2a1* 7 days after BSA treatment. **e**,**f**) Amputation wound cells do not up-regulate *Col10a1* 72 hours (**e**) or 7 days (**f**) after BSA treatment. **g**) In cavity forming digits 11 days after BMP9 treatment, cells in the center of the induced nodule immunostain positive for ColX (arrowhead) whereas cells in the stump are negative. **h**-**j**) BrdU incorporation studies **h**) Enhanced proliferation surrounding the BMP9 bead 72 hours after treatment in cavity forming digits, however cells of the induced nodule or cavity are not proliferating. **i**) Enhanced proliferation surrounding the BMP9 bead (*) 72 hours after treatment in non-cavity forming digits. **j**) Attenuated proliferation after BSA-treatment. **k**) *Prg4* transcripts are localized to cells undergoing cavitation during formation of the P1/P2 and P2/P3 synovial joints (arrowheads) at E18.5. **l**) *Prg4* is not expressed 72 hours after BSA treatment. **m**) *Prg4* is not expressed by

amputation wound cells after BMP9 bead treatment in non-cavity forming digits at 72 hours. **n**) The BMP9-induced joint-like structure is immunonegative for Dcx at 14 days. Adult articular cartilage is also immunonegative for Dcx. **o**) *Prg4* transcripts (arrowhead) are induced by cells of the amputation wound 72 hours after treatment with a BMP2 bead. **p**) Mallory trichrome staining of a B2-B9-treated cavity forming digit at 96 hours shows hypertrophic chondrocytes (arrow) associated with a stump endochondral response that is coupled with a regenerated cavity and a distal nodule. Distal is to the right and dorsal to the top. Scale bars: **a**-**f**, **h**-**m**,**o**=100µm; **g**=20 µm; **n**=50 µm.
