# Supplementary material for: BMP9 stimulates joint regeneration at digit amputation wounds in mice
Source: Nat Commun. 2019 Feb 5;10:424. doi: 10.1038/s41467-018-08278-4 (PMC6363752; doi:10.1038/s41467-018-08278-4)
Supplement: Supplementary file 3 — Reporting Summary [file 41467_2018_8278_MOESM3_ESM.pdf]

nature research

Corresponding author(s): Ken Muneoka

Reporting Summary

Nature Research wishes to improve the reproducibility of the work that we publish. This form provides structure for consistency and transparency in reporting. For further information on Nature Research policies, see Authors & Referees and the Editorial Policy Checklist.

Statistical parameters

When statistical analyses are reported, confirm that the following items are present in the relevant location (e.g. figure legend, table legend, main text, or Methods section).

n/a	Confirmed	
☐	☒	The exact sample size (n) for each experimental group/condition, given as a discrete number and unit of measurement
☐	☒	An indication of whether measurements were taken from distinct samples or whether the same sample was measured repeatedly
☐	☒	The statistical test(s) used AND whether they are one- or two-sided<br>*Only common tests should be described solely by name; describe more complex techniques in the Methods section.*
☒	☐	A description of all covariates tested
☐	☒	A description of any assumptions or corrections, such as tests of normality and adjustment for multiple comparisons
☐	☒	A full description of the statistics including central tendency (e.g. means) or other basic estimates (e.g. regression coefficient) AND variation (e.g. standard deviation) or associated estimates of uncertainty (e.g. confidence intervals)
☒	☐	For null hypothesis testing, the test statistic (e.g. F, t, r) with confidence intervals, effect sizes, degrees of freedom and P value noted<br>*Give P values as exact values whenever suitable.*
☒	☐	For Bayesian analysis, information on the choice of priors and Markov chain Monte Carlo settings
☒	☐	For hierarchical and complex designs, identification of the appropriate level for tests and full reporting of outcomes
☒	☐	Estimates of effect sizes (e.g. Cohen's d, Pearson's r), indicating how they were calculated
☐	☒	Clearly defined error bars<br>*State explicitly what error bars represent (e.g. SD, SE, CI)*

Our web collection on statistics for biologists may be useful.

Software and code

Policy information about availability of computer code

Data collection	We did not use software and code
Data analysis	Statistical analysis was performed using GraphPad PRISM (GraphPad Software, La Jolla,CA).

For manuscripts utilizing custom algorithms or software that are central to the research but not yet described in published literature, software must be made available to editors/reviewers upon request. We strongly encourage code deposition in a community repository (e.g. GitHub). See the Nature Research guidelines for submitting code & software for further information.

Data

Policy information about availability of data

All manuscripts must include a data availability statement. This statement should provide the following information, where applicable:

- Accession codes, unique identifiers, or web links for publicly available datasets
- A list of figures that have associated raw data
- A description of any restrictions on data availability

All relevant data are available from the authors upon request.

Field-specific reporting

Please select the best fit for your research. If you are not sure, read the appropriate sections before making your selection.

☒ Life sciences ☐ Behavioural & social sciences ☐ Ecological, evolutionary & environmental sciences

For a reference copy of the document with all sections, see nature.com/authors/policies/ReportingSummary-flat.pdf

Life sciences study design

All studies must disclose on these points even when the disclosure is negative.

Sample size	A minimum sample size of 8 was determined via power analysis (1-β = 0.8; α = 0.05). We used a sample size of ≥ 8 for all studies except for Prg4-/- mice treated with BSA, in which we used a sample size of 4 because there is no evidence that BSA induces joint regeneration in wildtype mice.
Data exclusions	No data was excluded from the study.
Replication	All experiments were replicated multiple times to ensure reproducibility. All attempts at replication were successful.
Randomization	Mice were allocated into experimental groups based on genotyping. Mice treated with growth factor or BSA-control was performed at random.
Blinding	Investigators were not blinded to group allocation during data collection and/or analysis as it was not relevant since the regenerative outcomes were anatomically based and compared to a highly reproducible failed regeneration response.

Reporting for specific materials, systems and methods

Materials & experimental systems

n/a	Involved in the study	
☒	☐	Unique biological materials
☐	☒	Antibodies
☐	☒	Eukaryotic cell lines
☒	☐	Palaeontology
☐	☒	Animals and other organisms
☒	☐	Human research participants

Methods

n/a	Involved in the study	
☒	☐	ChIP-seq
☒	☐	Flow cytometry
☒	☐	MRI-based neuroimaging

Antibodies

Antibodies used	Collagen 2: Acris Antibodies, San Diego, CA; AF5710; Alexa Fluor® 568 goat anti-mouse IgG: Invitrogen, Carlsbad, CA; AF11004; Aggrecan: EMD Millipore, Billerica, MA; AB1031; Alexa Fluor® 488 goat anti-mouse IgG Invitrogen, Carlsbad, CA; A11008; Doublecortin: Abcam, Cambridge, UK; AB207175; Collagen X: Abcam, Cambridge, UK; AB58632; Sox9, Abcam, Cambridge, UK; AB185966
Validation	The induced digit regeneration response has been well documented, and immunohistochemical staining has been described in the neonate (Yu et al. 2012; Lee et al. 2013) and adult (Dawson et al. 2017) responses. Starting with the manufacturer's recommended protocol, positive and negative control sections (e.g. anti-collagen 2 on a cartilage sample verses a muscle sample) are used to determine specificity, optimization of antigen retrieval method (no retrieval, heat-induced epitope retrieval, or proteinase K retrieval), and antibody dilution. Primary antibody detection utilizes secondary antibodies (Alexa Fluor® 488 or 568). Using Slidebook software (Intelligent Imaging Innovation Inc., Denver, CO) background subtraction of the autofluorescent signal is performed to ensure the integrity of the Alexa Fluor® signal, or imaging using multiple fluorescent filters was performed to identify background signal from authentic signal.

Eukaryotic cell lines

Policy information about cell lines

Cell line source(s)	BRITER BMP responsive cell line (Kerafast, Inc. Boston, MA).

Authentication	Dose response curve
Mycoplasma contamination	The cell lines were not tested for mycoplasma.
Commonly misidentified lines (See ICLAC register)	None were used.

Animals and other organisms

Policy information about studies involving animals; ARRIVE guidelines recommended for reporting animal research

Laboratory animals	Mus Musculus: CD1, male and female, 3 days old or 8 weeks old; Prg4 knockout mice (Prg4tm1Mawa/J) plus heterozygotes, male and female, 3 days old.
Wild animals	Wild animals were not used for these studies
Field-collected samples	Field-collected samples were not used for these studies
